# Stage-specific expression and divergent functions of two insulinase-like proteases associated with host infectivity in *Cryptosporidium*

**DOI:** 10.1371/journal.pntd.0012777

**Published:** 2025-01-13

**Authors:** Yue Huang, Shifeng Pei, Xin Lv, Fuxian Yang, Xiaoqing Gong, Na Li, Yaqiong Guo, Yaoyu Feng, Lihua Xiao

**Affiliations:** 1 State Key Laboratory for Animal Disease Control and Prevention, South China Agricultural University, Guangzhou, China; 2 Guangdong Laboratory for Lingnan Modern Agriculture, Center for Emerging and Zoonotic Diseases, College of Veterinary Medicine, South China Agricultural University, Guangzhou, China; Wadsworth Center, UNITED STATES OF AMERICA

## Abstract

**Background:**

The determinants of differences in host infectivity among *Cryptosporidium* species and subtypes are poorly understood. Results from recent comparative genomic studies suggest that gains and losses of multicopy subtelomeric genes encoding insulinase-like proteases (INS-19 and INS-20 in *Cryptosporidium parvum* and their orthologs in closely related species) may potentially contribute to these differences.

**Methodology/Principal findings:**

In this study, we investigated the expression and biological function of the INS-19 and INS-20 of *C*. *parvum*. CRISPR/Cas9 was used to endogenously tag both genes with the hemagglutinin epitope. Immunofluorescence analysis revealed that INS-19 and INS-20 are expressed at different developmental stages of the pathogen. Although knockout of either had no detectable effect on the *in vitro* growth of *C*. *parvum*, knockout of INS-20, deletion of its multiple domains, or mutation of the active motif in the functional domain reduced the intensity of *C*. *parvum* infection in IFN-γ knockout mice. Consistent with this, mice infected with the INS-20-deleted mutant had reduced intestinal damage and parasite burden.

**Conclusions/Significance:**

These results suggest that INS-19 and INS-20 have stage-specific expression with distinct biological functions, and that the presence of the INS-20 in zoonotic *C*. *parvum* contributes to its infectivity and fitness in mice.

## Author summary

Understanding why *Cryptosporidium* species and isolates differ in infectivity to their hosts is a complex puzzle. Recent comparative genomic analyses suggest that copy number variation of genes may be a contributing factor. In this research, we focused on two subtelomeric genes encoding the proteases *INS-19* and *INS-20* in *C*. *parvum*, the most important zoonotic *Cryptosporidium* species. We genetically tagged and engineered these genes to study their expression and function. Our results showed that INS-19 and INS-20 are expressed at different stages of the parasite life cycle. Interestingly, while knocking out each gene did not affect parasite growth *in vitro*, deleting or mutating INS-20 significantly reduced the severity of infection in mice. Mice infected with the *C*. *parvum* mutants had less intestinal damage and lower parasite loads. This study highlights the distinct roles of these two proteases and suggests that INS-20, in particular, plays a critical role in the parasite’s ability to infect and cause disease. These findings may facilitate the development of new drugs for cryptosporidiosis.

## Introduction

*Cryptosporidium* spp. are intracellular parasitic protozoa that cause cryptosporidiosis-associated diarrhea in humans and livestock. In low- and middle-income countries, cryptosporidiosis is highly prevalent and is the second leading cause of moderate-to-severe diarrhea in children under two years of age [1]. In high-income countries, cryptosporidiosis is a major food- and waterborne disease in various age groups [2]. In addition, *Cryptosporidium* spp. are prominent enteric pathogens affecting neonatal calves and lambs, causing diarrhea with significant mortality [3]. Of the nearly 50 named species, *Cryptosporidium hominis* and *Cryptosporidium parvum* are the most important in humans. *C*. *parvum* has a wide host range, including humans, nonhuman primates, cattle, sheep, horses, and rodents. Nearly 50% of human cases and most outbreaks of cryptosporidiosis in livestock are caused by this species [4]. In contrast, *C*. *hominis* primarily infects humans, although several divergent subtypes have recently been found in nonhuman primates and equine animals [5].

The genetic basis for the host infectivity of *Cryptosporidium* spp. is unclear. *C*. *parvum* and *C*. *hominis* were formerly the same species before being described as separate species [6]. They are ~96% identical in sequence at the genome level and share a nearly identical set of genes. However, these two species differ in the number of subtelomeric genes encoding insulinase-like proteases (INS), which are members of the M16 metalloprotease family. Although 98% of the genes in the small *Cryptosporidium* genome are single-copy, the INS protease family has 14–22 members in different species [7]. Previous studies suggest that these INS proteins are likely to be expressed in different organelles and thus play different roles in the life cycle. For example, the expression of INS-6 encoded by cgd2_4270 and INS-23 encoded by cgd5_3400 was recently localized to the microneme and mitosome of sporozoites, respectively, by spatial proteomics [8], while INS-16 encoded by cgd3_4270 was localized to the dense granules of sporozoites by immunoelectron microscopy [9]. In contrast, INS-1 is exclusively expressed in the macrogamete [10]. Deletion of INS-1 and neutralization of some INS proteins have been shown to inhibit *C*. *parvum* growth *in vitro* and *in vivo* [8–12].

Although most *INS* genes are located internally on chromosomes, exhibit high sequence divergence among members, and are shared by most enteric *Cryptosporidium* spp., the biological significance of INS-19 and INS-20 is highlighted by their distinct characteristics, which set them apart from other *Cryptosporidium* INS proteases. First, they share a high degree of sequence identity [11]. Other INS proteases are mostly identified by the presence of one or more M16 domains, but differ significantly in primary sequences. The only exceptions are INS-15 and INS-16, which have high sequence identity to each other, are encoded by paralogous genes, and share some sequence identity with INS-19 and INS-20 [9,13]. Second, the genes encoding INS-19 and INS-20 are located in the subtelomeric regions of chromosomes. A significant proportion of the subtelomeric genes are *Cryptosporidium*-specific, encoding proteins associated with the unique biological characteristics of *Cryptosporidium* spp. [14–16]. Third, the *INS-19* and *INS-20* genes are present only in *C*. *parvum*-related species [16]. Likely due to the subtelomeric location, gains and losses of these genes have been observed between different *Cryptosporidium* species and between *C*. *parvum* subtype families. For example, within *C*. *parvum*, the zoonotic IIa and IId subtypes possess both genes, while the human-adapted IIc subtype possesses only *INS-19*, and the rodent-adapted IIo and IIp subtypes possess only *INS-20* [17]. In contrast, both the INS-19 and INS-20 genes appear to be absent from the genomes of human *C*. *hominis* isolates sequenced to date [14,15,18–20]. Thus, these subtelomeric *INS* genes have been associated with the ability of different *C*. *parvum* subtype families to infect different hosts [14,21,22]. Fourth, as subtelomeric genes, there is a rapid evolution of sequences and some occurrence of genetic recombination, leading to the formation of phylogenetic clusters that are not separated by *Cryptosporidium* species [13–15].

In this study, we investigated the biological functions of the INS-19 (encoded by an *INS* gene located at the 3’ end of chromosome 6) and INS-20 (encoded by an *INS* gene located at the 5’ end of chromosome 5) in *C*. *parvum*. The genes encoding them in a *C*. *parvum* IId subtype were tagged with the hemagglutinin epitope for expression level and localization analysis. Their role in *C*. *parvum* growth and pathogenicity was evaluated by gene deletion and mutation. Modified lines with domain deletions and active residue mutations of the *INS-20* gene were constructed to identify specific functional sites.

## Materials and methods

### Ethics statement

The study was approved by the Laboratory Animal Center of South China Agricultural University (protocol # 2022c002). The animals used in the study were handled in accordance with the established guidelines for the use of laboratory animals in scientific research.

### Mice, parasite isolates and cell line

IFN-γ knockout (GKO) mice were used in the study. They were purchased from the Jackson Laboratory (Maine, USA) and bred in house. All mice were gender and age matched within individual experiments, and used at 3–4 weeks of age.

The *C*. *parvum* IIdA20G1-HLJ isolate was initially obtained from a dairy calf during a cryptosporidiosis outbreak [23], and maintained in the laboratory by serial passages in GKO mice as described [24]. This isolate was used because of its high infectivity, pathogenicity, and long duration of infection in GKO mice.

Human ileocecal adenocarcinoma (HCT-8) cells (ATCC CCL-244) were obtained from the Shanghai Branch of the Chinese Academy of Sciences and cultured in RPMI 1640 medium supplemented with 10% fetal bovine serum (FBS) and 1% penicillin-streptomycin solution at 37°C under 5% CO_2_.

### Bioinformatic analysis of INS-19 and INS-20

Domains in INS-19 and INS-20 sequences were predicted using the InterPro software (https://www.ebi.ac.uk/interpro/). The 3D structures of these proteases were constructed using the software AlphaFold 2.3.2 (https://deepmind.google/technologies/alphafold/). The genetic relationship of INS-19 and INS-20 protein sequences from different *Cryptosporidium* species and *C*. *parvum* subtypes was assessed using the neighbor-joining analysis implemented in MEGA 5 (https://www.megasoftware.net/), with 1000 replicates of bootstrapping.

### *C*. *parvum* mutant construction

The Nluc-Neo selection cassette from a previous study was used to genetically modify the *INS-19* and *INS-20* genes in *C*. *parvum* [25]. To tag the two subtelomeric *INS* genes of the IId subtype, gRNAs targeting the 3’ end of the gene were used. For this, the 5’ and 3’ flanking genomic regions were amplified by PCR as left and right homologous arms, respectively, to tag the *INS-19* and *INS-20* genes with a 3× hemagglutinin (HA) epitope [26]. To knock out the *INS-19* and *INS-20* genes, a double sgRNA strategy targeting the 5’ and 3’ regions of the genes was used [10], and sequences flanking the gene were used as homologous arms for recombination. To delete the domain of the *INS-20* gene, gRNAs targeting the 5’ end were used to knock out the M16 active domain (nucleotides 261–585) or to mutate the enzymatic site of *INS-20*. In contrast, gRNA targeting the 3’ end of the gene was used to delete the C-terminal sequence (nucleotides 2076–3384). To use nanoluciferase as a surrogate for parasite load, a control line was constructed using a gRNA targeting the 3’ end of the thymidine kinase (*TK*) gene [27]. The sequences upstream and downstream of the gRNA were used to integrate a GFP fluorescent tag and the Nluc-Neo selection cassette downstream of the *TK* gene. The primers used in this study were synthesized by Integrated DNA Technologies (Tsingke Biotech, Beijing, China) and are listed in S1 Table.

### Isolation of oocysts and transfection of sporozoites

Fecal samples were collected daily from mice infected with wildtype *C*. *parvum*. Oocysts were purified from fecal material using sucrose and cesium chloride gradient centrifugation [28].

To obtain sporozoites, oocysts were resuspended in 600 μL of PBS and treated with 200 μL of bleach (Clorox, California, USA) on ice for 10 min. After three washes with PBS by centrifugation, sodium taurocholate (Sigma, New Jersey, USA) was added to the oocyst suspension to a final concentration of 0.75%. The oocyst suspension was incubated at 37°C for 60 min, and the sporozoites released were harvested by centrifugation at 16,000 g for 3 min and resuspended at the concentration of 5 x 10^7^ sporozoites/80 μL of SF buffer (Lonza, Basel, Switzerland).

For the transfection of sporozoites, donor and CRISPR/Cas9 plasmids (50 μg in 100 μL each) were added to the sporozoite suspension in a 100-μL electroporation cuvette (Lonza). The sporozoites were electroporated using the EH100 program on an AMAXA 4D Nucleofector System (Lonza).

### Selection and amplification of transgenic parasites

To obtain genetically modified lines of the *C*. *parvum* isolates, two GKO mice were infected with 2.5 x 10^7^ electroporated sporozoites five min after the neutralization of gastric acid by oral gavage with 200 μL of 8% sodium bicarbonate solution. Paromomycin (16 g/L) was added to the drinking water of the mice 18 h post infection (HPI). The establishment of the transgenic lines was verified using fecal luciferase assay. Oocysts were purified from positive fecal samples as described above.

### Verification of correct integration of replacement templates

PCR was used to initially verify correct integration of the replacement cassettes. The PCR reaction consisted of 1 μL DNA extracted from fecal pellets using the Omega Stool DNA Kit (Omega, Georgia, USA), Phanta Max PCR Buffer, the Phanta Max Super-Fidelity DNA Polymerase (Vazyme, JiangSu, China), and the primers listed in S1 Table. The PCR reaction was performed on a Veriti 96-well thermal cycler (Bio-Rad, California, USA), and the PCR products were identified by electrophoresis of 1.0% agarose gel containing GelRed (1:10,000). The successful deletion of the *INS-19* and *INS-20* genes was confirmed by whole-genome sequencing of DNA extracted from purified oocysts using the 150-bp paired-end Illumina technology as described [29]. The resulting sequence reads were mapped to the reference *C*. *parvum* genome (NCBI No. GCA_030415345.1) using bwa-mem2 and Samtools [21]. The generated bam and index files were imported into the IGV software [30] to visualize the gene deletion by checking the coverage of the target gene by the sequence reads in the reference *C*. *parvum* genome.

### Indirect immunofluorescence microscopy

Oocysts were excysted as described above and the resulting sporozoites were placed on SuperStick slides (Waterborne, Inc., New Orleans, USA). Asexual stages of *C*. *parvum* were obtained from HCT-8 cells were cultured on coverslips at 24 HPI, while the sexual stages were obtained from HCT-8 cultures at 48 HPI. The slides and coverslips were fixed with 4% paraformaldehyde (Leagene, Beijing, China), treated with 0.3% Triton X-100 (Sigma, New Jersey, USA), and blocked with 3% bovine serum albumin (BSA). They were incubated first with the rabbit anti-HA tag antibody (Thermo) at 1:100 dilution, then with Alexa Fluor 488-conjugated goat-anti-rabbit IgG (CST, Boston, USA), and finally with Sporo-Glo (Waterborne, Inc.). Hoechst (Thermo) was used to stain cell nuclei. The slides and coverslips were examined under a BX53 fluorescence microscope (Olympus, Tokyo, Japan).

### In vitro proliferation assay

HCT-8 cells were cultured in a 48-well cell culture plate to 95% confluence. They were infected with IId-GFP, *Δins-19*, and *Δins-20* lines at 1×10^4^ oocysts per well. Infected cells were harvested at 3, 24, and 48 HPI for luciferase assay.

### Determination of parasite load by luciferase assay

Nanoluciferase assay was performed using the Nano-Glo Luciferase Assay kit (Promega, Wisconsin, USA). For the analysis of cell cultures, the luciferase activity was determined as previously described [26]. For the analysis of fecal samples, 100 mg of fecal pellets and 3-mm glass beads (Thermo, Massachusetts, USA) were added to 1 mL of PBS in microfuge tubes. After thorough vortex, 50 μL of the supernatant was transferred to a white 96-well ELISA plate and mixed with 50 μL of luminescence reagent. The luminescence was measured using a Multi-Mode Reader (BioTek, Vermont, USA).

### Assessment of gene deletion on host infectivity

To study the biological function of the *INS* genes, GKO mice (N = 6 per group) were infected with the IId-GFP, Δ*ins-19*, and Δ*ins-20* lines. To further evaluate the contribution of functional domains to the biological function of INS-20, GKO mice were infected with the IId-GFP, INS-20ΔNter, INS-20ΔCter, and INS-20^HLLEQ/5A^ lines (N = 3 per group). The inoculation dose in both studies was 1×10^3^ oocysts/mouse. All mice were housed separately in individual cages. Body weight was recorded for each mouse every two to three days, while fecal samples were collected from infected mice every six days for measurement of oocyst shedding using a luciferase assay. The linear relationship between fecal luminescence activity and oocysts per gram of feces, as determined by qPCR analysis of the SSU rRNA gene [31], was established by comparative analysis of infection of GKO mice with the IId-GFP line.

### Histological examination and scanning electron microscopy

Six GKO mice were infected with 1×10^3^ oocysts of IId-GFP, *Δins-19*, or *Δins-20* lines (N = 2 per group). Nine days post infection (DPI), the mice were euthanized, and the ileum was fixed with 10% buffered formalin (Leagene, Beijing, China) for hematoxylin and eosin (H&E) staining, and with 2.5% glutaraldehyde for scanning electron microscopy. For H&E microscopy, the villus-to-crypt height ratio and the number of oocysts per villus were determined for 25 villi. For scanning electron microscopy, the tissue sections were subjected to critical point drying and gold sputtering using conventional methods. The coated sample was examined under an EVO MA 15/LS 15 scanning electron microscope (Carl Zeiss, Oberkochen, Germany).

### Statistical analysis

All statistical analyses were performed using Graphpad Prism (California, USA). Unpaired *t-*test was used to compare the means of two groups in histological examination, Mann-Whitney U test was used to compare the means of two groups in *in vitro* proliferation, Gehan-Breslow-Wilcoxon test was used to analyze the survival data of the mice. Differences with *p* values ≤ 0.05 were considered significant.

## Results

### INS-19 and INS-20 contain multiple domains and different active motifs

**Fig 1:**
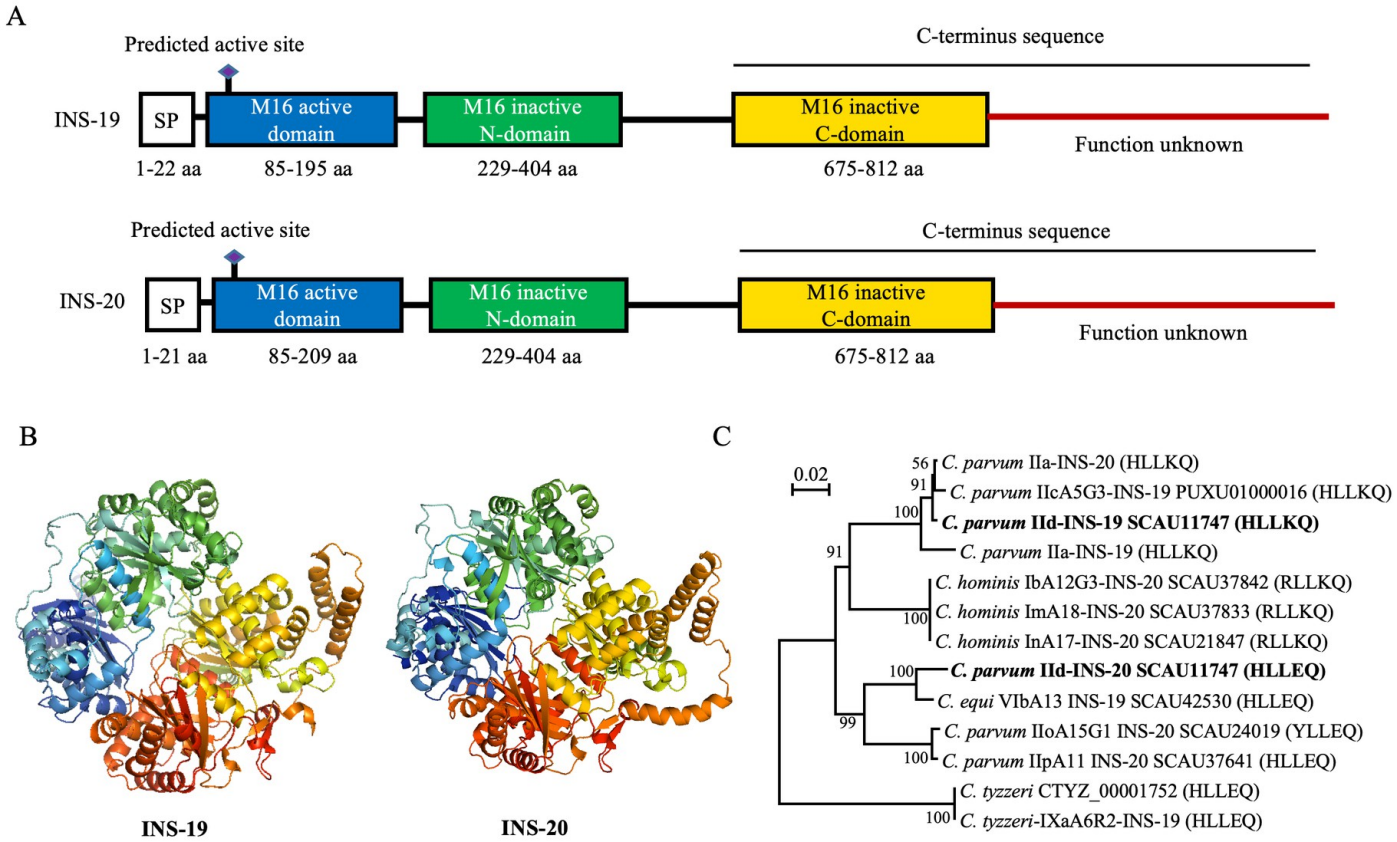
Sequence characteristics of INS-19 and INS-20 from several *Cryptosporidium* species. (A) Domains of INS-19 and INS-20 sequences predicted by InterPro software. INS-19 and INS-20 possess a signal peptide, an active M16 domain containing a zinc-binding motif, an inactive M16 domain at the N-terminus, and an inactive M16 domain at the C-terminus. (B) 3D structures of INS-19 and INS-20 predicted by AlphaFold. INS-19 and INS-20 each consist of four distinct structural components. (C) Phylogenetic relationship of INS-19 and INS-20 from several *Cryptosporidium* spp. based on neighbor-joining analysis of amino acid sequences, with *Cryptosporidium tyzzeri* as the outgroup. INS-19 and INS-20 sequences from multiple *Cryptosporidium* spp. form several clusters that are not completely segregated by species. Sequences in parentheses represent the predicted active motif within the active domain. Numbers on nodes are percent bootstrap values from 1000 replicate analyses.

InterPro analysis of the translated amino acid sequences revealed that INS-19 (NCBI No. PP909794) and INS-20 (NCBI No. PP909795) of the *C*. *parvum* IIdA20G1-HLJ isolate both contain a signal peptide and an active M16 domain at the N-terminus, two inactive M16 domains, and a sequence of unknown function at the C-terminus (Fig 1A). However, the AlphaFold2 prediction of the 3D structure of INS-19 and INS-20 revealed the presence of four distinct domains, consistent with the structure of classical M16 proteases (Fig 1B).

When the amino acid sequences of several *Cryptosporidium* spp. were compared, the INS-19 and INS-20 proteases were polymorphic, with sequence differences ranging from 0% to 28.2%. Most of the sequence differences were in the C-terminus, but they also had different motif sequences of the active site within the active M16 domain, including ’HLLEQ’, ’HLLKQ’, ’YLLEQ’ and ’RLLKQ’. Among the *C*. *parvum* subtypes analyzed, IIa-INS-19, IIc-INS-19, IId-INS-19, and IIa-INS-20 had the ’HLLKQ’ sequence, while IId-INS-20 and IIp-INS-20 had the ’HLLEQ’ sequence. In addition, the INS-19 of *C*. *equi* and *C*. *tyzzeri* sequences analyzed had the HLLEQ’ sequence, while the INS-20 of nonhuman primate-adapted *C*. *hominis* had the ’RLLKQ’ sequence (Fig 1C).

In the neighbor-joining analysis of these amino acid sequences, the INS-19 and INS-20 proteases from several *Cryptosporidium* spp. formed two major groups with two clades each. In particular, the INS-19 and INS-20 of the IIa subtype clustered together, whereas those of the IId subtype were placed in two separate clusters. Furthermore, the Ib-INS-20, Im-INS-20, and In-INS-20 of *C*. *hominis* from macaque monkeys were identical to each other and formed a clade between the two major clades formed by *C*. *parvum* (Fig 1C). In general, INS-19 and INS-20 sequences with the same active motif were grouped together in the same clade. For example, IIa-INS-19, IIc-INS-19, IId-INS-19, and IIa-INS-20, which all had the ’HLLKQ’ sequence, clustered together. Similarly, IId-INS-20 and INS-19 of *C*. *equi*, both with the ’HLLEQ’ sequence, formed a clade distant from the above cluster (Fig 1C).

### INS-19 and INS-20 have stage-specific expression

**Fig 2:**
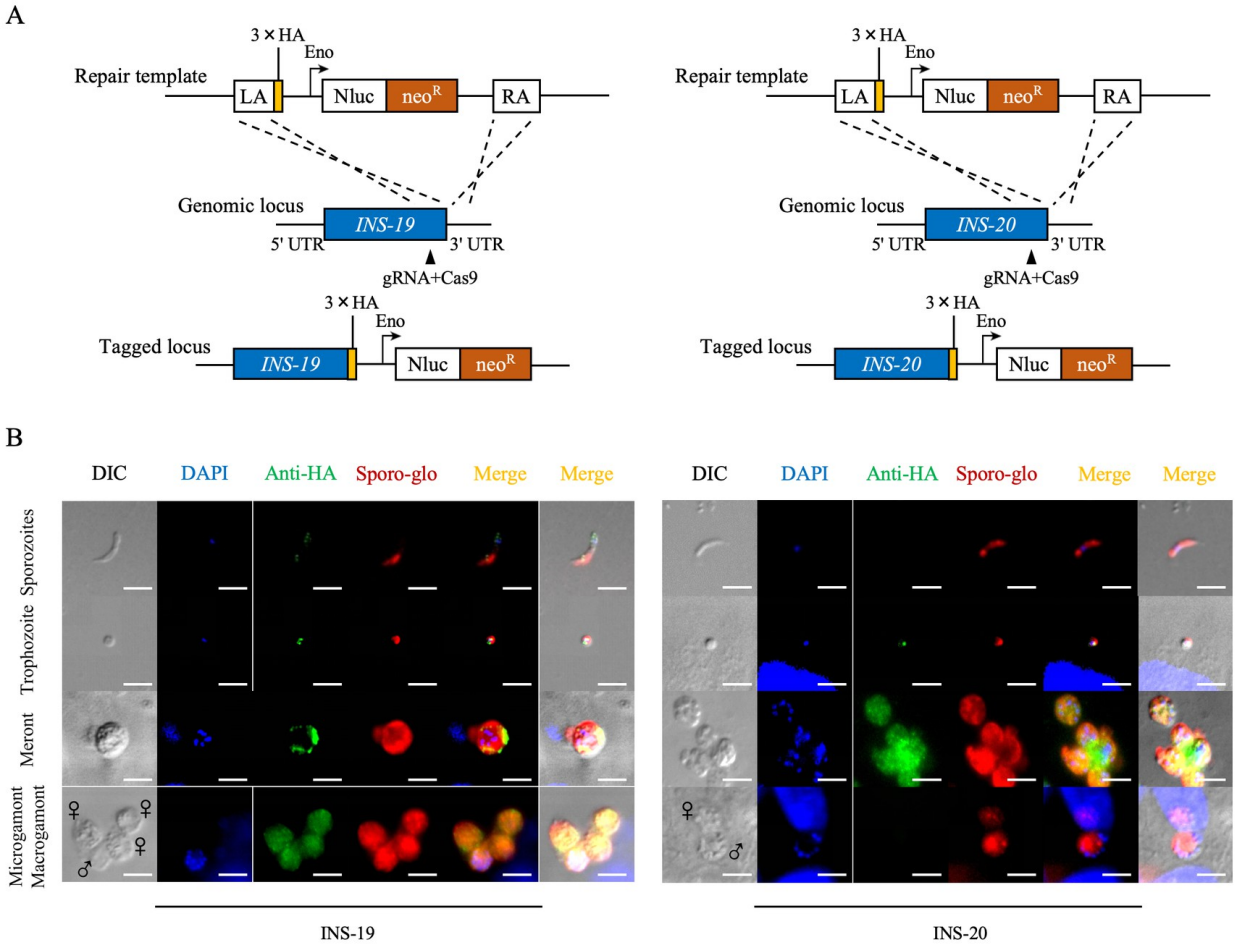
Expression profiles of INS-19 and INS-20 in *Cryptosporidium parvum* based on endogenous tagging of the genes. (A) Illustrations of endogenous tagging of the *INS-19* and *INS-20* genes with a 3× HA tag and the Nluc-P2A-Neo sequence at the C-terminus. (B) Expression patterns of INS-19 and INS-20 in transgenic lines as indicated by immunofluorescence microscopy of developmental stages using a mAb against the HA tag, with the Sporo-glo (a polyclonal antibody against developmental stages of *C*. *parvum*) and the Hoechst (a nuclear stain) as controls. INS-19 and INS-20 have different expression patterns during the developmental stages in *C*. *parvum*. Scale bars = 5 μm.

CRISPR/Cas9 was used to tag the INS-19 and INS-20 proteases in a *C*. *parvum* IId isolate with the 3× hemagglutinin epitope (3HA) at the C-terminus (Fig 2A). PCR analysis of the 5’ and 3’ ends of the genetically modified locus in the feces of GKO mice inoculated with the transgenic line yielded PCR products of the expected size (S1A Fig). In IFA analysis of parasite stages using a mAb against the HA tag, the asexual stages of *Cryptosporidium* were distinguished from the sexual stages by culture time and morphology; in this study, the sexual stages were analyzed at 48 DPI, macrogametes had no visible nuclei, whereas microgamonts contained more than 8 nuclei. INS-19 expression showed a punctate distribution within sporozoites, trophozoites, meronts, microgamonts, and macrogametes (Figs 2B and S2A). In contrast, the punctate distribution of INS-20 was restricted to trophozoites and meronts (Figs 2B and S2B). These results suggest that INS-19 and INS-20 may play multiple and distinct roles in different developmental stages of *C*. *parvum*.

### *INS-19* and *INS-20* are dispensable

A double sgRNA strategy was used to knock out the *INS-19* and *INS-20* genes using CRISPR/Cas9 technology (S1B Fig). PCR analysis of fecal samples from infected mice showed that the Nluc-neo selection cassette was correctly inserted into the gene deletion locus, and PCR primers targeting the *INS-19* and *INS-20* CDS failed to generate a product from the knockout line (S1B Fig). Furthermore, whole-genome sequencing of *Δins-19* (NCBI No. SRR29366321) and *Δins-20* (NCBI No. SRR29366320) transgenic lines confirmed that the target gene was completely removed (S1B Fig). The results indicated that we have successfully generated *Δins-19* and *Δins-20* transgenic lines, and that these two genes are dispensable. In addition, we constructed a IId-GFP line (S3A Fig) to serve as a control for the two knockout lines. To avoid affecting the parasite load or virulence of the transgenic lines after mouse infection, a GFP tag and a Nluc-neo selection cassette were inserted downstream of the *TK* gene without altering its expression. Nanoluciferase activity in the feces of mice infected with the IId-GFP line correlated linearly with oocyst shedding intensity measured by qPCR analysis of DNA extracted from the same samples. Thus, nanoluciferase activity was used as a convenient indicator of parasite burden (S3B Fig).

### *INS-19* and *INS-20* knockout reduces parasite shedding in mice

**Fig 3:**
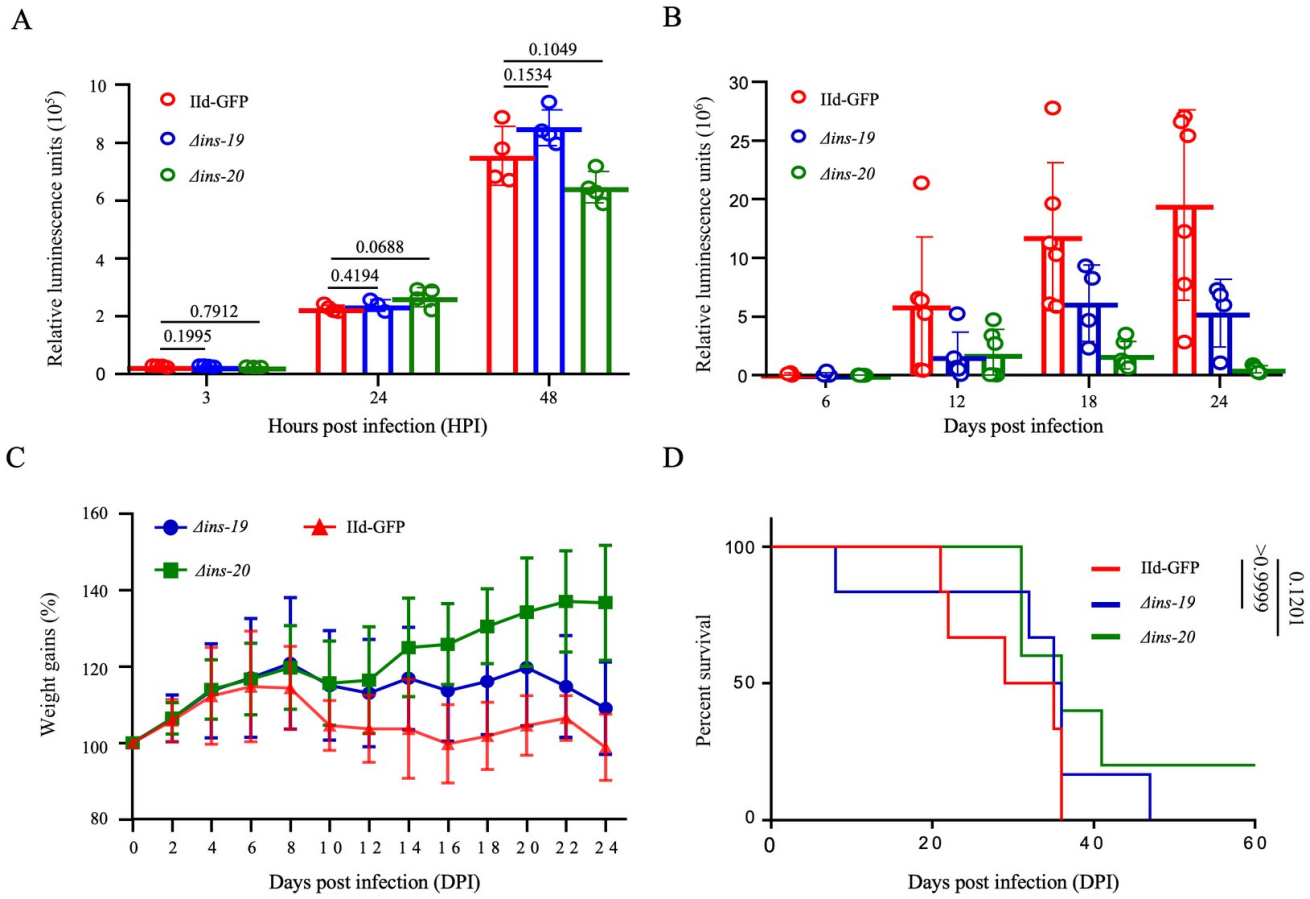
Effect of *INS-19* and *INS-20* gene knockout on the growth of *Cryptosporidium parvum*. (A) Growth patterns of the IId-GFP, *Δins-19* and *Δins-20* lines in HCT-8 cell cultures. Differences between groups are not significant (*p =* 0.1995, *p =* 0.4194, *p =* 0.1534 for *Δins-19* at 3, 24, and 48 HPI, respectively; and *p =* 0.7912, *p =* 0.0688, *p =* 0.1049 for *Δins-20* at 3, 24, and 48 HPI, respectively). N = 4. Bars are standard deviations. (B) Oocyst shedding patterns in GKO mice infected with IId-GFP, *Δins-19* and Δ*ins-20* lines as indicated by fecal luciferase activity. At the peak of infection, mice infected with the *Δins-19* line showed a threefold decrease in fecal luciferase activity compared to those infected with the IId-GFP line, while those infected with the *Δins-20* line showed a 6-27-fold decrease in fecal luciferase activity. N = 6. Bars are standard deviations. (C) Differences in weight gain of GKO mice after infection with *Δins-19*, Δ*ins-20* and IId-GFP lines. Compared to the mice infected with the IId-GFP line, mice infected with the *Δins-20* line had higher body weights. N = 6. Bars are standard deviations. (D) Survival rates of the GKO mice infected with IId-GFP, *Δins-19* and *Δins-20* lines. No significant differences in survival rates were observed between the groups (*p >* 0.9999 and *p =* 0.1201 for *Δins-19* and *Δins-20*, respectively).

To evaluate the effect of *INS-19* or *INS-20* gene deletion on *Cryptosporidium* growth and development, we compared the growth of *Δins-19*, *Δins-20*, and IId-GFP lines in HCT-8 cells. The results showed that the *Δins-19* and *Δins-20* lines grew at a comparable rate to the IId-GFP line in HCT-8 cultures at 3, 24 and 48 HPI (Fig 3A). This suggests that INS-19 and INS-20 are not required for parasite invasion and growth *in vitro*.

To evaluate the importance of INS-19 or INS-20 in *C*. *parvum* infectivity, GKO mice were infected with 10^3^ oocysts from genetically modified lines and compared with the IId-GFP line for oocyst shedding intensity, body weight gain and survival rates. The results showed that the mean luciferase activity in fecal samples from mice infected with the *Δins-19* line was 3-fold lower at DPI 24. In contrast, mice infected with the *Δins-20* line showed a 6-27-fold reduction in luciferase activity during DPI 18–24 (Fig 3B). In addition, the body weight of the mice infected with *Δins-20* increased after infection, while those infected with the IId-GFP and *Δins-19* lines showed no weight gain (Fig 3C). However, there were only insignificant differences in the survival of infected mice between these groups. In particular, GKO mice infected with the *Δins-20* line showed slight delay in death (Fig 3D).

### *INS-19* and *INS-20* knockout reduces intestinal injury and parasite burden in mice

**Fig 4:**
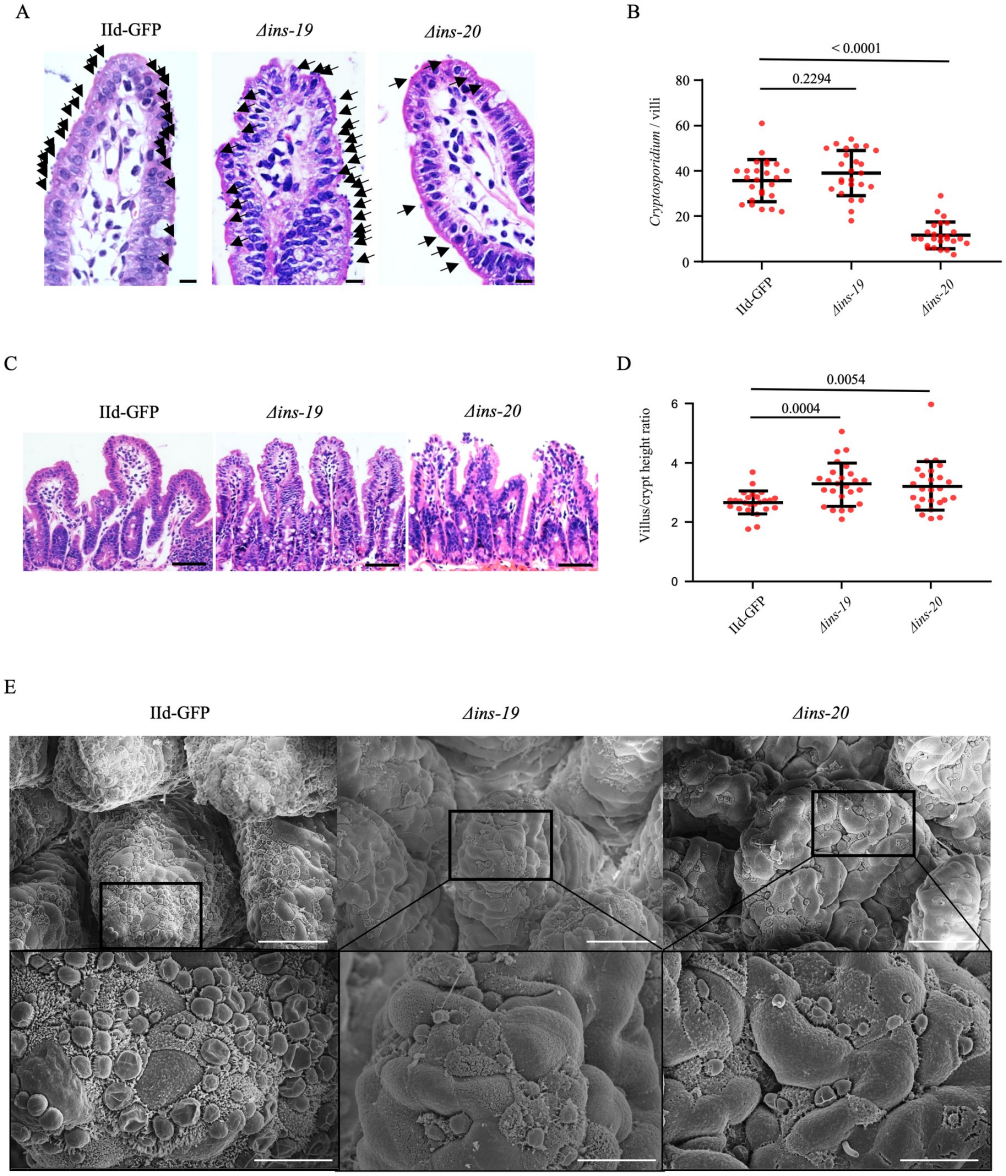
Differences in histological changes and parasite load of intestinal tissues among mice infected with *Δ*ins-19, *Δ*ins-20, and IId-GFP lines of *Cryptosporidium parvum*. (A) Hematoxylin and eosin microscopy images of the ileum from GKO mice infected with IId-GFP, *Δins-19* and *Δins-20* lines taken at high magnification. Scale bars = 10 μm. (B) Parasite load in the intestine from GKO mice infected with IId-GFP, *Δins-19* and *Δins-20* lines. The *INS-20* gene knockout significantly reduces the *Cryptosporidium* load (*p =* 0.2294 and *p <* 0.0001 for Δ*ins-19* and Δ*ins-20*). N = 25. Bar indicates standard deviation. (C) Hematoxylin and eosin microscopy images of the ileum of GKO mice infected with IId-GFP, *Δins-19* and *Δins-20* lines taken at low magnification. Scale bars = 50 μm. (D) The ratio of villus to crypt depth of GKO mice infected with IId-GFP, *Δins-19* and *Δins-20* lines. Knockout of *INS-19* or *INS-20* genes attenuates the pathological damage in the mouse intestine (*p =* 0.0004 and *p =* 0.0054 for *Δins-19* and *Δins-20*). N = 25. Bar indicates standard deviation. (E) *Cryptosporidium* load in the intestines of GKO mice infected with IId-GFP, *Δins-19* and *Δins-20* lines using scanning electron microscopy. The absence of each reduces the *Cryptosporidium* load. For the top three images, scale bar = 30 μm, for the bottom three images, scale bar = 10 μm.

Histological analysis of the ileum revealed that a lower parasite load was observed in the ileum of mice infected with the *Δins-20* line than those in mice infected with the *Δins-19* and IId-GFP lines (Figs 4A, 4B and S4A). In addition, the ratio of intestinal villus length to crypt depth was significantly reduced in mice infected with the IId-GFP line compared to those infected with the *Δins-19* and *Δins-20* lines likely due to the overall lower parasite load (Figs 4C, 4D and S4B). Scanning electron microscopy of ileal tissues further confirmed the low parasite load on the intestinal epithelium in mice infected with the *Δins-19* and *Δins-20* lines, especially the latter (Fig 4E).

### *INS-20* requires proper structure and active motif to function

**Fig 5:**
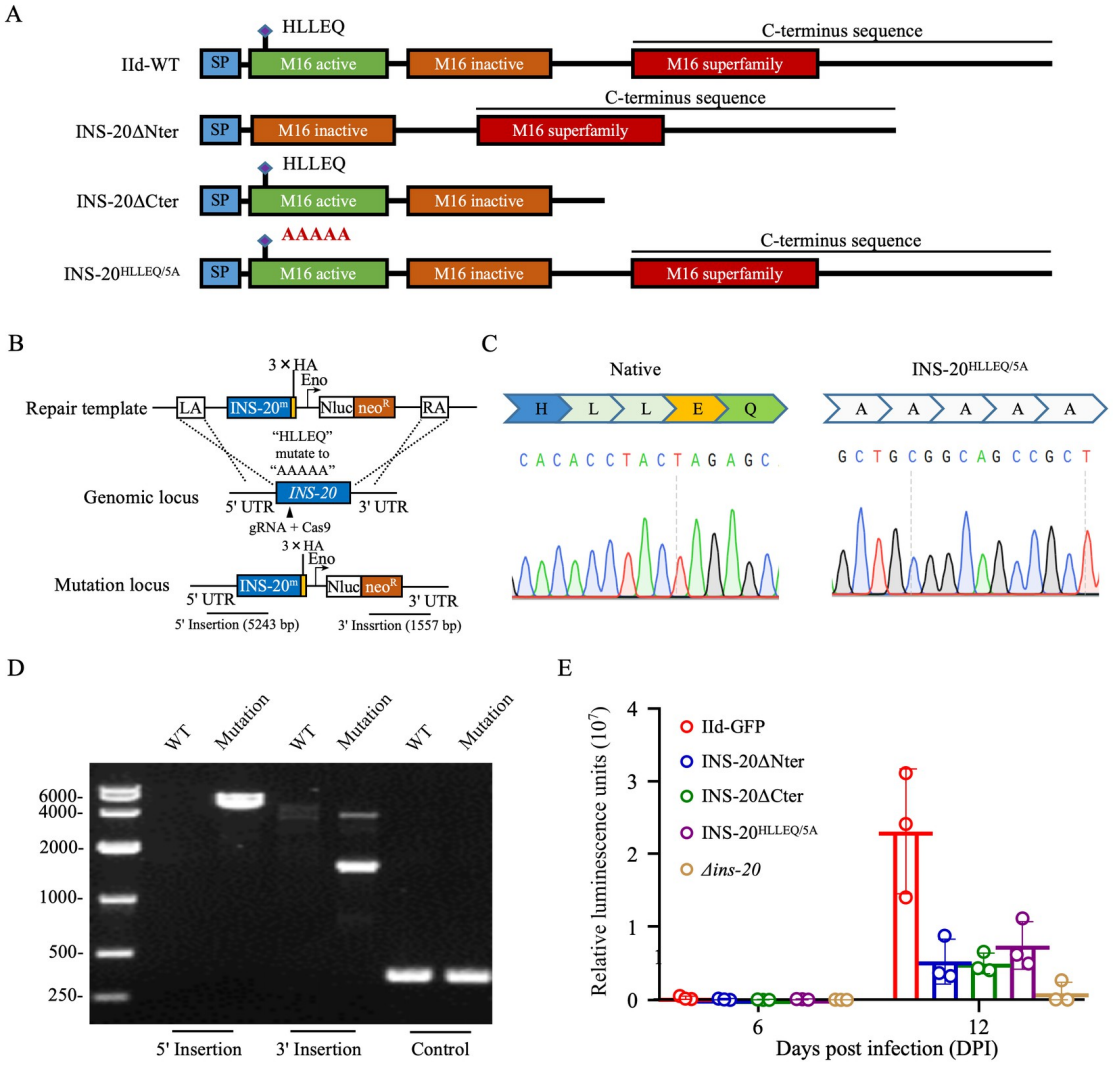
Effect of deletion of the functional domains of INS-20 on *Cryptosporidium parvum*. (A) Illustration of the native and modified loci within the *INS-20* gene for WT, INS-20ΔNter, INS-20ΔCter, and INS-20^HLLEQ/5A^ lines. (B) Illustration of the mutation of the functional motif ’HLLEQ’ within the active domain of the INS-20 to ’AAAAA’. (C) Sequence analysis of PCR products from native INS-20 (left) and mutant INS-20^HLLEQ/5A^ (right). (D) Nucleic acid electrophoresis analysis of the mutation of the motif ’HLLEQ’ within the active domain of the INS-20 to ’AAAAA’. The PCR products "5’ Insertion" and "3’ Insertion" confirm correct integration. The "Control" product is specific for the INS3 locus. (E) Oocyst shedding patterns in GKO mice infected with INS-20ΔNter, INS-20ΔCter, and INS-20^HLLEQ/5A^ and IId-GFP lines as indicated by fecal luciferase activity. Mice infected with the transgenic lines of INS-20ΔNter, INS-20ΔCter, and INS-20^HLLEQ/5A^ showed a 3-fold decrease in fecal luciferase activity compared to those infected with the IId-GFP line. N = 3, bar indicates standard deviation.

Since deletion of *INS-20* greatly reduced the parasite load in GKO mice, we evaluated the effect on parasite load by deleting the N-terminal active domain and the C-terminal sequence of *INS-20*, and mutating the predicted active motif ’HLLEQ’ to ’AAAAA’ to identify the sequence that is critical for the biological function of INS-20 (Fig 5A). PCR and sequencing analysis confirmed the accurate editing of the target sequences (Figs 5B–5D and S1C). IFA analysis showed that INS-20 retained its expression and intracellular localization after the knockout of these domains or mutation of the amino acids (S5 Fig). In mouse infection studies (N = 3 per group), the luciferase activity per gram of feces in GKO mice infected with the INS-20ΔNter, INS-20ΔCter, and INS-20^HLLEQ/5A^ lines was 4–5 times lower than that in mice infected with the IId-GFP line at peak oocyst shedding (DPI 12) (Fig 5E).

## Discussion

The results of this study suggest distinct roles for INS-19 and INS-20 in host infectivity of *C*. *parvum*. Based on bioinformatics analysis, *INS-19* and *INS-20* genes from several *Cryptosporidium* species and subtypes share high sequence identity and possess 4 domains that are present in M16 proteases, suggesting conservation in 3D structures. However, endogenous gene tagging has revealed distinct expression patterns of INS-19 and INS-20 in *C*. *parvum*, suggesting diversification of INS functions despite the high sequence identity. This is supported by the results of gene knockout studies. While knockout of INS-19 and INS-20 individually did not significantly affect the *in vitro* growth of *C*. *parvum*, a marked reduction in infection intensity was observed in GKO mice following knockout of the *INS-20* gene or its domains. These results demonstrate that the presence of INS-20 in *C*. *parvum* enhances its infectivity in mice.

It is unlikely that INS-19 and INS-20 function as classical M16 metalloproteases. Classical M16 proteases such as human insulinase have four domains, with two N-terminal domains and two C-terminal domains that independently form the inner substrate-binding chamber [32, 33]. Notably, only three domains were found in INS-19 and INS-20 in the InterPro analysis, with the inactive M16 domain at the C-terminus missing. However, using 3D modeling with AlphaFold, these proteases were found to have four domains. Therefore, the four domains of INS-19 and INS-20 may form an internal substrate-binding chamber that is distinct from classical M16 metalloproteases.

The divergence in the function of INS-19 and INS-20 from classical M16 metalloproteases is also supported by the sequence differences in the functional motif within the active M16 domain. In classical M16 metalloproteases, the active motif for zinc binding has the sequence ’HxxEH’ (where x can be any amino acid) [34]. However, INS-19 as well as IIa-INS-19, IIa-INS-20, and IIc-INS-19 have the sequence ’HLLKQ’, while INS-20 has the sequence ’HLLEQ’ in the active M16 domain. This suggests that INS-19 and INS-20 have evolved functions distinct from those of classical M16 proteases. In *C*. *parvum*, of the 22 INS proteins, only INS-1, INS-4, INS-15 and INS-16 have been shown to have four classical M16 domains with the active motif sequence of ’HxxEH’ [9, 10, 12, 13, 35]. Other *C*. *parvum* INS proteins are much smaller in size and contain only 1–3 M16 domains. It has been suggested that some of them form dimers to function [12, 13].

Although INS-20 has a natural mutation in a key residue of the zinc-binding motif of M16 proteases, this sequence appears to be necessary for its function. Our study showed that mutation of the ’HLLEQ’ motif in INS-20 disrupts its biological functions with reduced oocyst shedding of *C*. *parvum* in GKO mice. Previous studies have confirmed the importance of the ’HxxEH’ motif in M16 metalloproteases, which is critical for zinc binding and hydrolysis of substrate peptide bonds [36, 37]. In light of this, we propose that INS-20 may function by slightly different mechanisms than typical M16 metalloproteases. This implies that despite sequence variations, INS-20 may catalyze similar reactions or participate in different biological processes.

As with classical M16 metalloproteases, the maintenance of a proper 3D structure appears to be necessary for the biological function of INS-20. In this study, we individually knocked out the N-terminal M16 active domain and the C-terminal sequence of INS-20. The results showed that these genetic modifications significantly reduced *C*. *parvum* infectivity in GKO mice, suggesting that, similar to classical M16 metalloproteases, INS-20 may also require these sequences to form an internal substrate-binding chamber and thereby exert its biological functions.

Despite the high sequence identity, the expression profiles of INS-19 and INS-20 in *C*. *parvum* differ from each other. Results from endogenous gene tagging indicate that INS-19 has a broader expression range than INS-20. It is expressed in most developmental stages of *C*. *parvum* with the exception of immature meronts (4N). In contrast, INS-20 is mainly expressed in meronts (4N and 8N). These results suggest that INS-19 and INS-20 may have different roles in *C*. *parvum*. A previous study of INS-19 using polyclonal antibodies showed the expression of INS-19 in all developmental stages of *C*. *parvum* [11]. This may be due to antibody reactivity to sequences shared by INS-19 and INS-20.

The results of the gene deletion studies support the biological importance of INS-19 and INS-20 and the functional differences between them. Although knockout of either *INS-19* or *INS-20* genes did not significantly affect the *in vitro* proliferation of the *C*. *parvum*, loss of either *INS-19* or *INS-20* reduced the intestinal parasite load and pathogenicity of *C*. *parvum* in GKO mice. The effect of *INS-20* knockout was more significant than that of *INS-19* knockout. This suggests that INS-20 plays a more important role than INS-19 in host infectivity of *C*. *parvum*. Previously, the absence of the *INS-19* and *INS-20* genes in the genomes of many *Cryptosporidium* species was associated with their narrow host range [14, 21]. Therefore, they may be potential candidates for the development of alternative vaccines against *C*. *parvum*. Recently, a vaccine targeting the GP40 glycoprotein has been developed against *C*. *parvum* [38]. Since GP40 is a variant surface protein that functions very differently from INS-19 and INS-20 [39], the inclusion of other targets in the newly developed vaccine would broaden the target range (e.g. from IIa subtypes to IIc and IId subtypes) and provide an additional layer of protection.

In conclusion, the results of this study support the contribution of INS-19 and INS-20 to host infectivity and fitness of *C*. *parvum*. They appear to function differently from other *Cryptosporidium* INS proteins due to their unique structure and functional motif sequence. These sequence differences may contribute to the differences in the expression profiles and biological significance between INS-19 and INS-20. Further studies are needed to verify the applicability of these findings to immunocompetent mice and other hosts, to identify the subcellular localization and substrates of these enzymes, and to characterize the molecular processes underlying their diverse functions.

## Supporting information

S1 Fig Illustrations of the *Cryptosporidium parvum* loci modified in this study and confirmed by PCR analysis or whole-genome sequencing. (A) Endogenous tagging of the *INS-19* and *INS-20* genes. The presence of "5’ Insertion" and "3’ Insertion" PCR products confirms correct integration. The "Control" PCR product is specific for the INS3 locus. (B) Knockout of the *INS-19* and *INS-20* genes using a double sgRNA strategy. The PCR product "5’ Insertion" and "3’ Insertion" confirm correct integration. The PCR product "CDS" corresponds to fragments of the *INS-19* and *INS-20* open reading frames that are only detectable in the wild type (WT). The "Control" PCR product is specific for the INS3 locus. DNA from *Δins-19* and *Δins-20* lines was subjected to whole-genome sequencing. Individual sequence reads mapping to *INS-19* or *INS-20* and surrounding loci are shown. Note gene loss. (C) Knockout of the N-terminal M16 active domain and the C-terminal sequence in the *INS-20* gene. The PCR products "5’ Insertion" and "3’ Insertion" confirm correct integration. The PCR product "CDS" corresponds to fragments of the *INS-20* open reading frame, detectable with different sizes in the WT and INS-20ΔNter lines, but not in the INS-20ΔCter line. The "Control" product is specific for the INS3 locus.
(TIF)

S2 Fig Expression time analysis of INS-19 and INS-20 in *Cryptosporidium parvum*. **The expression patterns of INS-19 and INS-20 in transgenic lines were analyzed using immunofluorescence microscopy at different developmental stages.** A monoclonal antibody against the HA tag was used for detection, with Sporo-glo and Hoechst serving as controls. The results showed distinct expression patterns: INS-19 was widely expressed, including both sexual and asexual stages, whereas INS-20 was mainly expressed during the asexual stages in *C*. *parvum*. Scale bars = 5 μm.
(TIF)

S3 Fig Establishment of a linear relationship between parasite load measured by luminescence (x-axis) and qPCR (y-axis) using the IId-GFP line of *Cryptosporidium parvum*. (A) Construction of IId-GFP lines by insertion of a GFP cassette downstream of the *TK* gene in *C*. *parvum*. Green fluorescence was observed in the IId-GFP line under fluorescence microscopy. Scale bars = 10 μm. (B) Oocyst shedding patterns in GKO mice infected with the IId-GFP line measured by luminescence (x-axis) and qPCR (y-axis). Each measurement represents the average of three technical replicates from the six fecal samples, and the linear relationship has an R^2^ value of 0.98.
(TIF)

S4 Fig Hematoxylin and eosin microscopy images of the ileum from GKO mice infected with IId-GFP, *Δins-19* and *Δins-20* lines of *Cryptosporidium parvum*. (A) Images of the ileum from GKO mice infected with IId-GFP, *Δins-19* and *Δins-20* lines taken at high magnification. Scale bars = 10 μm. (B) Images of the ileum of GKO mice infected with IId-GFP, *Δins-19* and *Δins-20* lines taken at low magnification. Scale bars = 50 μm.
(TIF)

S5 Fig Immunofluorescence analysis of INS-20 in INS-20ΔNter, INS-20ΔCter, and INS-20^HLLEQ/5A^ lines at the trophozoite and meront stages using an mAb against the HA tag, with the Sporo-glo (a polyclonal antibody against developmental stages of *C*. *parvum*) as a control. Scale bars = 5 μm. The results indicate that the deletion of domains or point mutations at key amino acid sites do not prevent expression of the remaining INS-20 sequence.
(TIF)

S1 Table Oligonucleotides used in this study. (DOCX)

S1 Data Raw data for figures in this study. (XLSX)
