# Supplementary figures and images for: Stage-specific expression and divergent functions of two insulinase-like proteases associated with host infectivity in Cryptosporidium
Source: PLoS Negl Trop Dis. 2025 Jan 13;19(1):e0012777. doi: 10.1371/journal.pntd.0012777 (PMC11760560; doi:10.1371/journal.pntd.0012777)

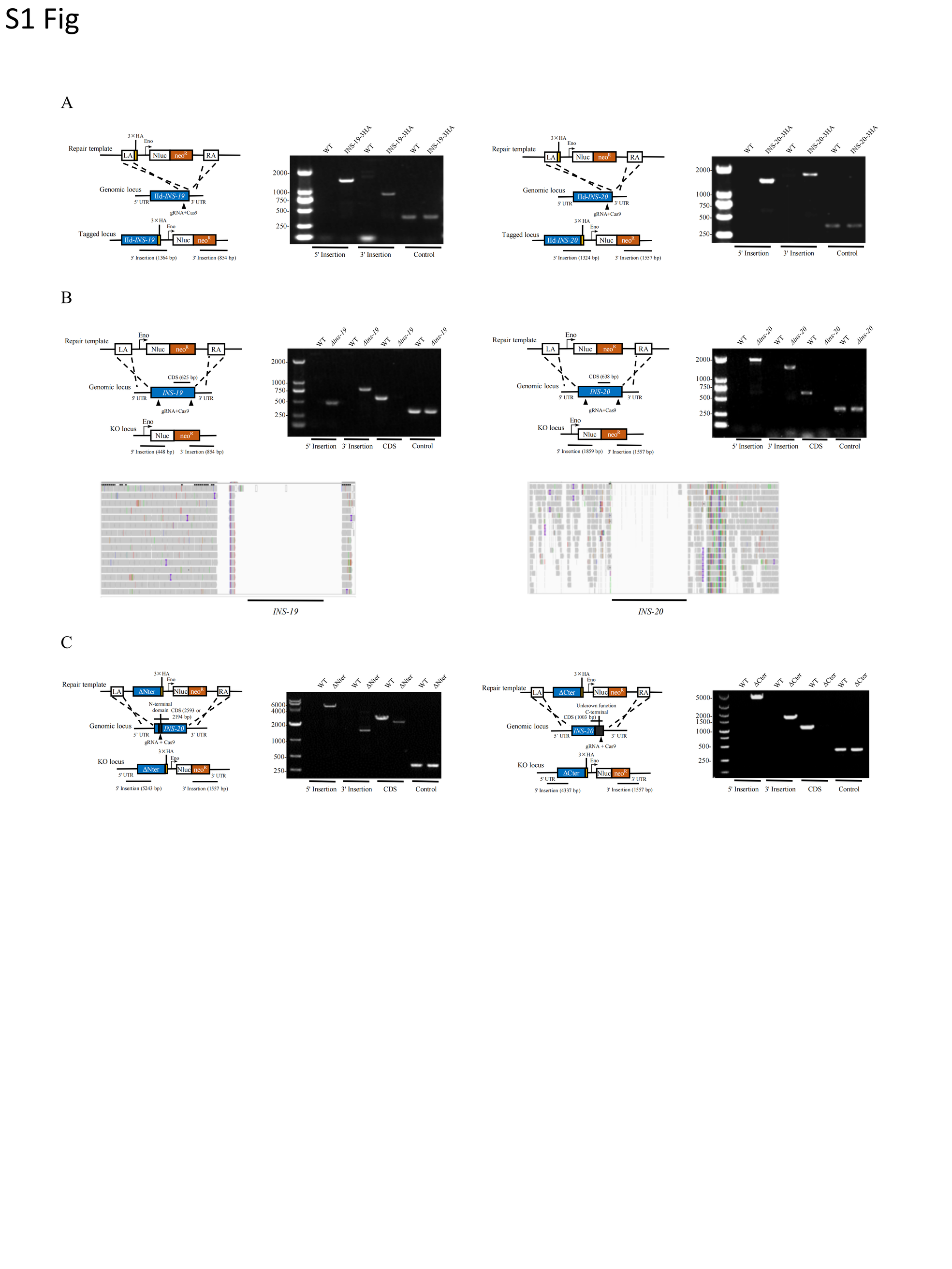

Supplement: S1 Fig — (A) Endogenous tagging of the INS-19 and INS-20 genes. The presence of "5’ Insertion" and "3’ Insertion" PCR products confirms correct integration. The "Control" PCR product is specific for the INS3 locus. (B) Knockout of the INS-19 and INS-20 genes using a double sgRNA strategy. The PCR product "5’ Insertion" and "3’ Insertion" confirm correct integration. The PCR product "CDS" corresponds to fragments of the INS-19 and INS-20 open reading frames that are only detectable in the wild type (WT). The "Control" PCR product is specific for the INS3 locus. DNA from Δins-19 and Δins-20 lines was subjected to whole-genome sequencing. Individual sequence reads mapping to INS-19 or INS-20 and surrounding loci are shown. Note gene loss. (C) Knockout of the N-terminal M16 active domain and the C-terminal sequence in the INS-20 gene. The PCR products "5’ Insertion" and "3’ Insertion" confirm correct integration. The PCR product "CDS" corresponds to fragments of the INS-20 open reading frame, detectable with different sizes in the WT and INS-20ΔNter lines, but not in the INS-20ΔCter line. The "Control" product is specific for the INS3 locus. (TIF) [file pntd.0012777.s001.tif]

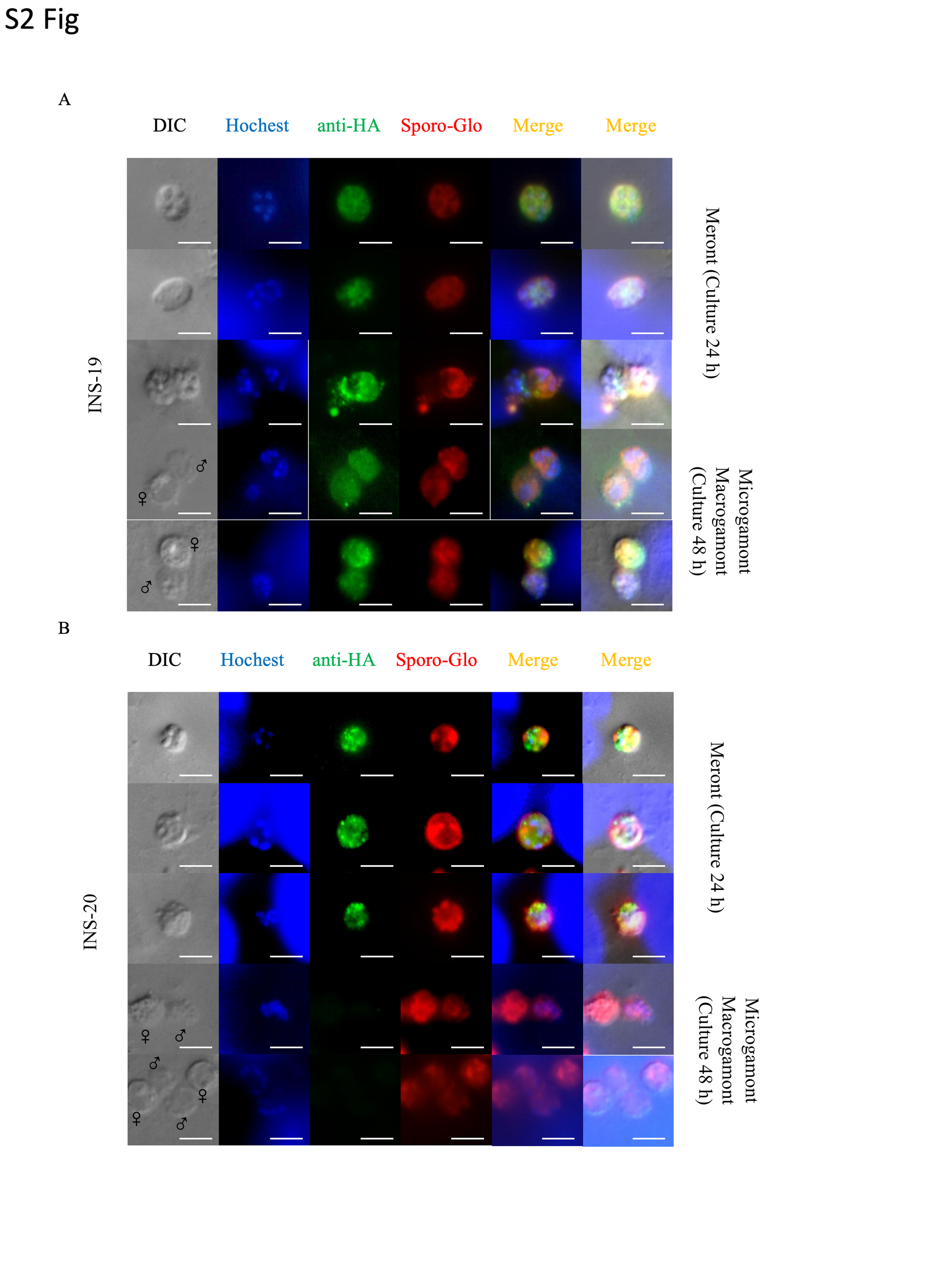

Supplement: S2 Fig — The expression patterns of INS-19 and INS-20 in transgenic lines were analyzed using immunofluorescence microscopy at different developmental stages. A monoclonal antibody against the HA tag was used for detection, with Sporo-glo and Hoechst serving as controls. The results showed distinct expression patterns: INS-19 was widely expressed, including both sexual and asexual stages, whereas INS-20 was mainly expressed during the asexual stages in C. parvum. Scale bars = 5 μm. (TIF) [file pntd.0012777.s002.tif]

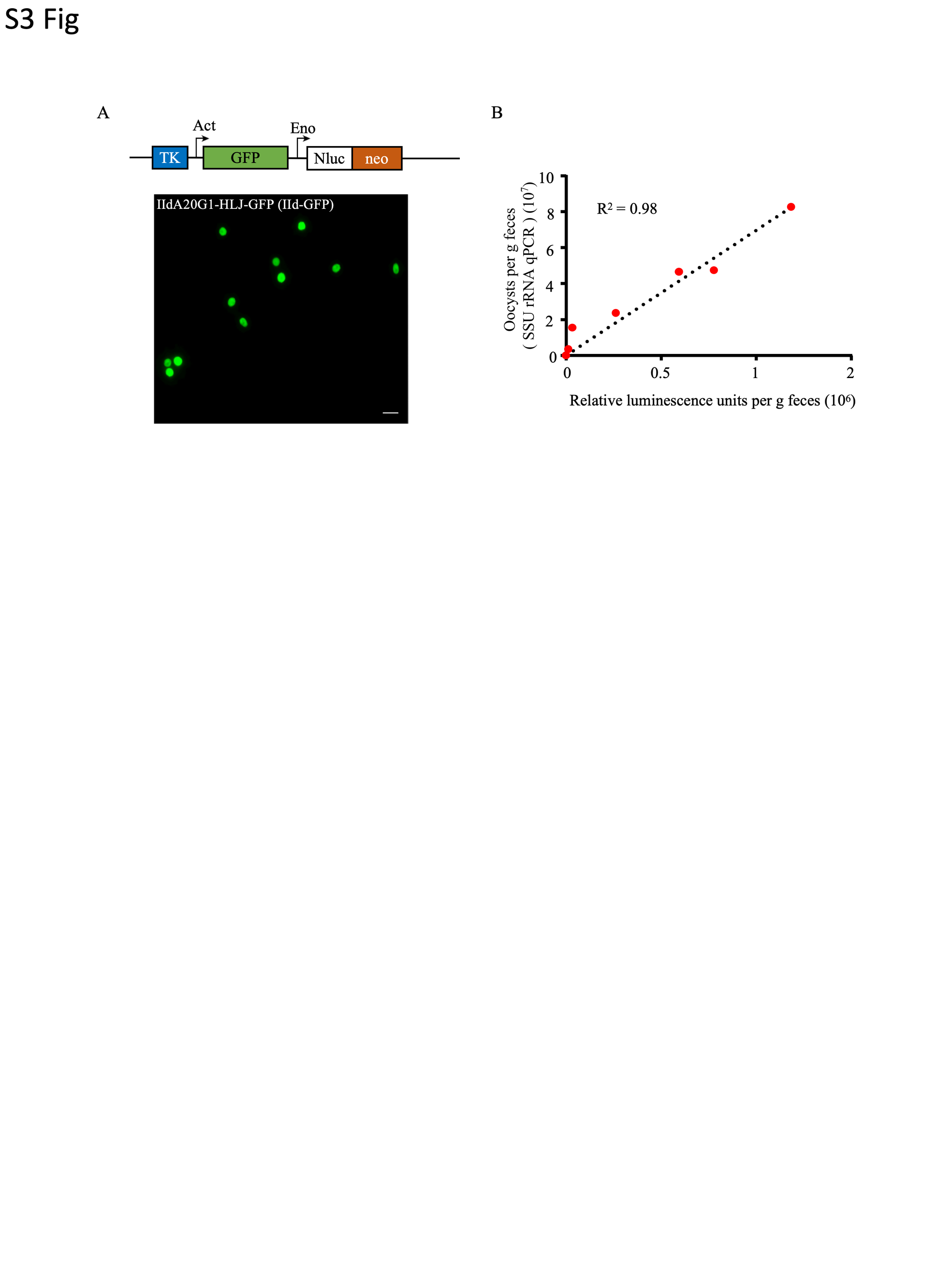

Supplement: S3 Fig — (A) Construction of IId-GFP lines by insertion of a GFP cassette downstream of the TK gene in C. parvum. Green fluorescence was observed in the IId-GFP line under fluorescence microscopy. Scale bars = 10 μm. (B) Oocyst shedding patterns in GKO mice infected with the IId-GFP line measured by luminescence (x-axis) and qPCR (y-axis). Each measurement represents the average of three technical replicates from the six fecal samples, and the linear relationship has an R2 value of 0.98. (TIF) [file pntd.0012777.s003.tif]

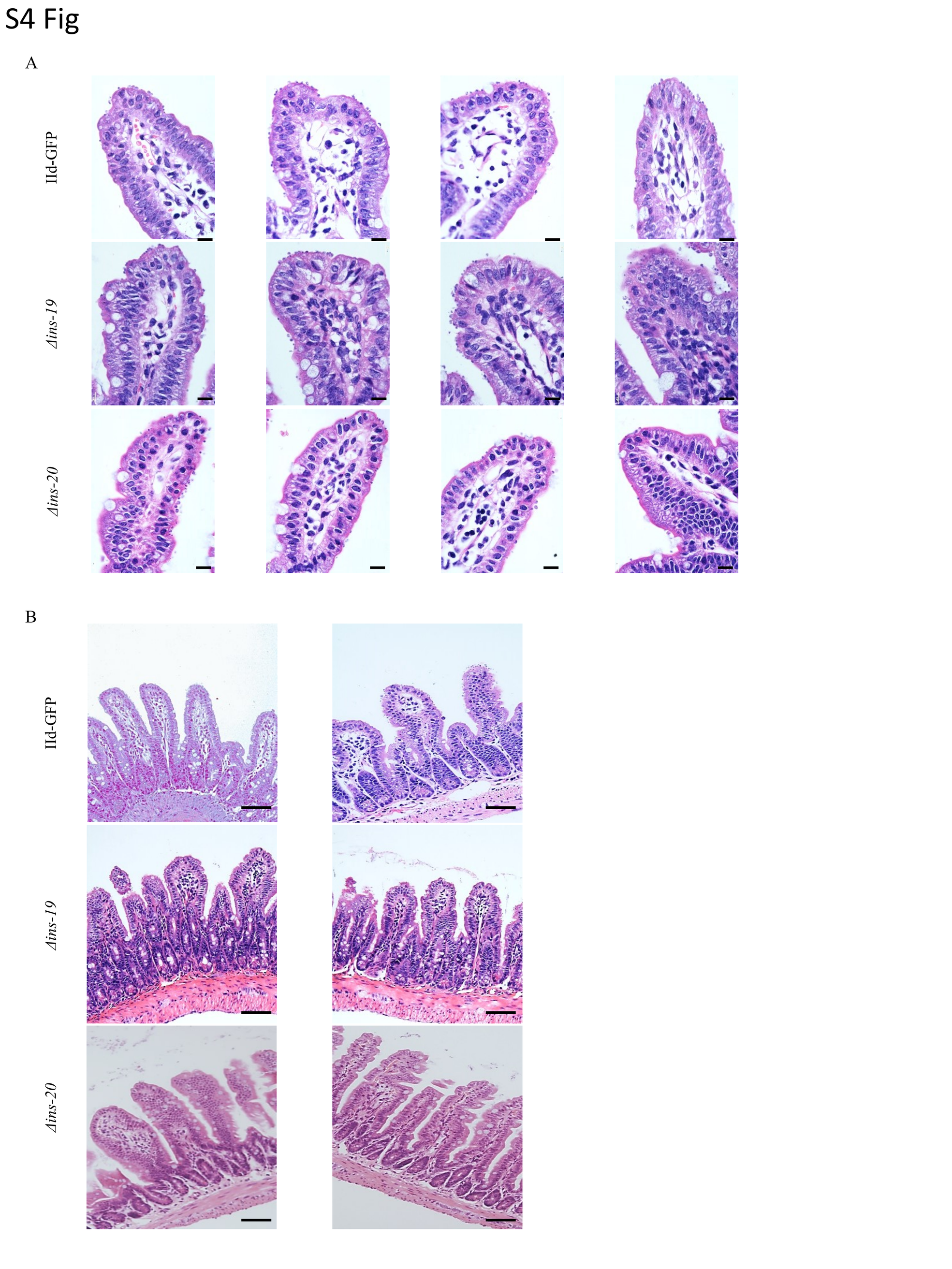

Supplement: S4 Fig — (A) Images of the ileum from GKO mice infected with IId-GFP, Δins-19 and Δins-20 lines taken at high magnification. Scale bars = 10 μm. (B) Images of the ileum of GKO mice infected with IId-GFP, Δins-19 and Δins-20 lines taken at low magnification. Scale bars = 50 μm. (TIF) [file pntd.0012777.s004.tif]

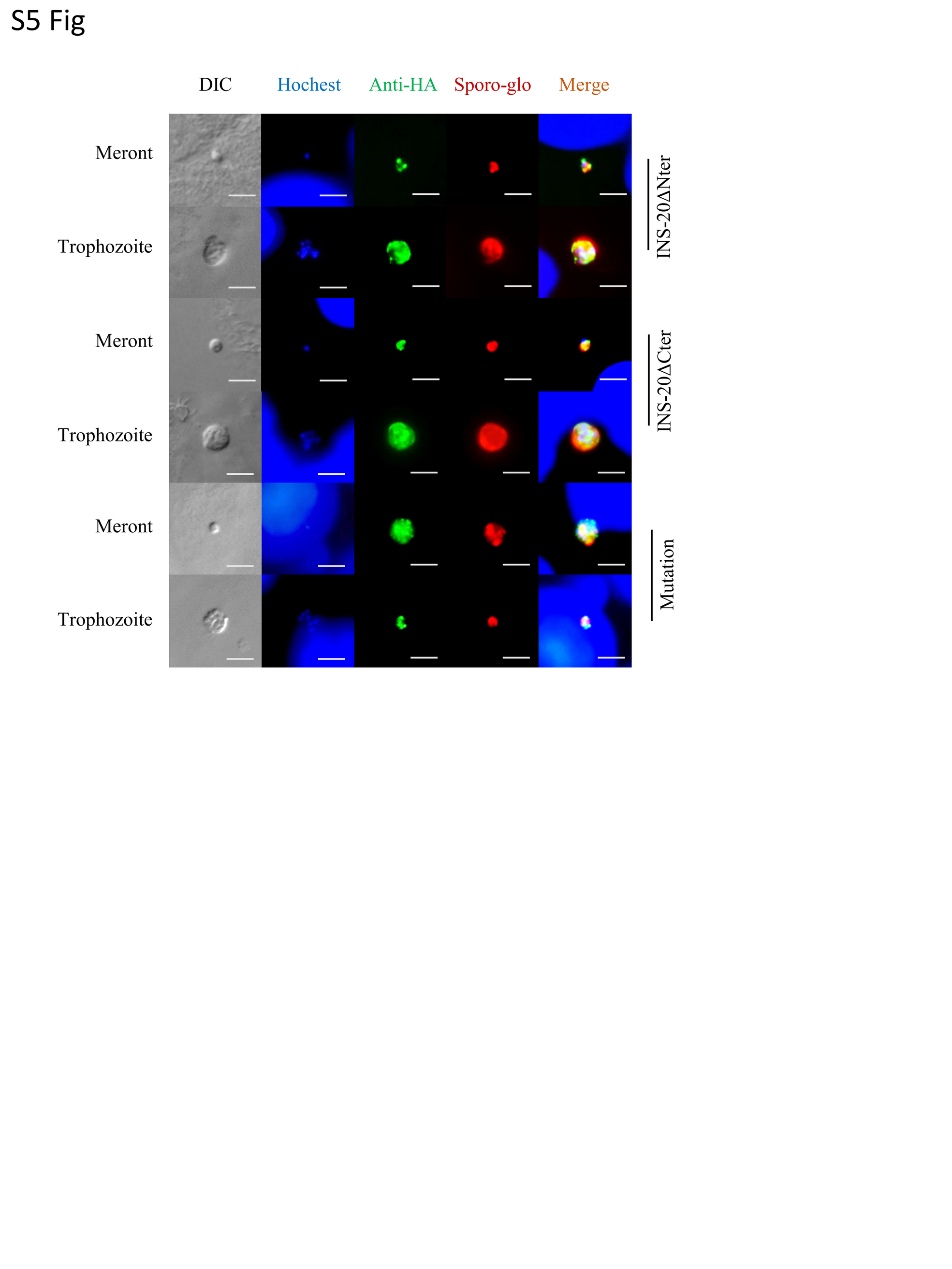

Supplement: S5 Fig — Scale bars = 5 μm. The results indicate that the deletion of domains or point mutations at key amino acid sites do not prevent expression of the remaining INS-20 sequence. (TIF) [file pntd.0012777.s005.tif]
