# Supplementary material for: Stage-specific expression and divergent functions of two insulinase-like proteases associated with host infectivity in Cryptosporidium
Source: PLoS Negl Trop Dis. 2025 Jan 13;19(1):e0012777. doi: 10.1371/journal.pntd.0012777 (PMC11760560; doi:10.1371/journal.pntd.0012777)
Supplement: S1 Table — (DOCX) [file pntd.0012777.s006.docx]

| Table S1. Oligonucleotides used in this study. | | | | |
| --- | --- | --- | --- | --- |
| **Usage** | **Oligo name** | **Sequence (5’ – 3’)** | **Specific purpose** | **Source** |
| **IId-Tagging** | INS-19-C ter-F | AGTGAATTCGACCTGAGCTCCTCAACTGGATGAAGTATTTTATAATGAAAATAATATCG | Amplify INS-19 C-terminus homology arm (548 bp) | This study |
|  | INS-19-C ter-R | AGCCCGAGCCCTTGCTAGCCTTATCTTTCATGAATTTGAGTTGGTTATATGT |  |  |
|  | INS-19-3UTR-F | GTCGAAATCTAACTCGGAGTTTTATAACATTTGTTTAAAATCATTACCTC | Amplify INS-19 3'UTR homology arm (424 bp) | This study |
|  | INS-19-3UTR-R | GTCGACTCTAGAGGATCTCCATTAAGCATAAATTCACTTAAATTATTTTTCCTTG |  |  |
|  | INS-20-C ter-F | GTGAATTCGAGCTGAGCTCATCAGGTAAACTCACAAGTATTACAATCTATTATTTG | Amplify INS-20 C-terminus homology arm (590bp) | This study |
|  | INS-20-C ter-R | AGCCCGAGCCCTTGCTAGCCTCATCTTTCATGAATTTGAGTTGGTTATATGC |  |  |
|  | INS-20-3UTR-F | GTCGAAATCTAACTCGGGGTATTTCAATTAATATATTTACCTTCGTTAAAATC | Amplify INS-20 3'UTR homology arm (257 bp) | This study |
|  | INS-20-3UTR-R | GTCGACTCTAGAGGATCTCCAGTTGTCGGAAATATCATAGTTTATAGGT |  |  |
|  | lacZ F2 | GGGGATCCTCTAGAGTCGAC | Amplify plasmid backbone (2684 bp) | [10] |
|  | lacZ R1 | GGGTACCGAGCTCGAATT |  |  |
|  | Linker-F1 | GCTAGCAAGGGCTCGGGC | Amplify 3HA-Nluc-P2A-neo cassette (2215 bp) | This study |
|  | Terminator-R | CCGAGTTAGATTTCGACTTTATAATGAACAAG |  |  |
|  | INS-19-PAM-F | CTCTTCATTCAACAAGTTCATGGAGTGGTTTGAACAAAT | Mutate PAM sequence of INS-19 gRNA | This study |
|  | INS-19-PAM-R | TGAACTTGTTGAATGAAGAGTCAATAGAAAGCCAGCTGTTAAAATG |  |  |
|  | INS-20-PAM-F | TACGTGGTCACCACGATAGCTGGTTTTCTATTAAATTATTATCCT | Mutate PAM sequence of INS-19 gRNA | This study |
|  | INS-20-PAM-R | GCTATCGTGGTGACCACGTATATTTTTTTTAAGAATTTTGTAATTAAAATCATG |  |  |
|  | tracr RNA-F1 | GTTTTAGAGCTAGAAATAGCAAG | Amplify Cas9 plasmid backbone (9963 bp) | [10] |
|  | U6 R | CCCAACACTTAACCTTTCAGT |  |  |
|  | INS-19-gRNA(C)-linker | CTATTTCTAGCTCTAAAACGCGATTAAATAGTACTATTCCCCAACACTTAACCTTTCA | Building pACT1:Cas9-GFP, U6:sgINS-19(C) | This study |
|  | INS-20-gRNA(C)-linker | TGAAAGGTTAAGTGTTGGGGAATAGTACTATTTAATCGCGTTTTAGAGCTAGAAATAG | Building pACT1:Cas9-GFP, U6:sgINS-20(C) |  |

Table S1. Oligonucleotides used in this study. (continued)

| **Usage** | | **Oligo name** | | **Sequence (5’ – 3’)** | **Specific purpose** | | **Source** | |
| --- | --- | --- | --- | --- | --- | --- | --- | --- |
| **IId-Knockout** | | INS-19-5'UTR-F1 | AGTGAATTCGAGCTGAGCTCGCATTTTATAATCTGTTTAATCCAATCATG | | Amplify INS-19 5'UTR homology arm (247 bp) | This study |  |  |
|  |  | INS-19-5'UTR-R1 | TATATTTAGTTTCCCCACCTCATTTTGTTACTTTTACAGTGACATTTAAG | |  |  |  |  |
|  |  | INS-19UTR-F2 | GTCGAAATCTAACTCGGAGTTTTATAACATTTGTTTAAAATCATTACCTC | | Amplify INS-19 3'UTR homology arm (424 bp) | This study |  |  |
|  |  | INS-19UTR-R2 | GTCGACTCTAGAGGATCTCCATTAAGCATAAATTCACTTAAATTATTTTTCCTTG | |  |  |  |  |
|  |  | INS-20-5'UTR-F1 | AGTGAATTCGAGCTGAGCTCCAACTTGGATATACTGTTCGTACACAAGAAG | | Amplify INS-20 5'UTR homology arm (1156 bp) | This study |  |  |
|  |  | INS-20-5'UTR-R1 | TATATTTAGTTTCCCCACCTGTTAGATGTACAATCTATTTGACTTTAAGGC | |  |  |  |  |
|  |  | INS-20-UTR-F2 | GTCGAAATCTAACTCGGGGTATTTCAATTAATATATTTACCTTCGTTAAAATC | | Amplify INS-20 3'UTR homology arm (900 bp) | This study |  |  |
|  |  | INS-20-UTR-R2 | GTCGACTCTAGAGGATCTCCAGTTGTCGGAAATATCATAGTTTATAGGT | |  |  |  |  |
|  |  | Eno-F | AGGTGGGGAAACTAAATATACTGAAATTCG | | Amplify Nluc-P2A-neo cassette (1879 bp) | This study |  |  |
|  |  | Terminator-R | CCGAGTTAGATTTCGACTTTATAATGAACAAG | |  |  |  |  |
|  |  | INS-19-gRNA(N)-linker | CTATTTCTAGCTCTAAAACGCGATTAAATAGTACTATTCCCCAACACTTAACCTTTCA | | Building pACT1:Cas9-GFP, U6:sgINS-19(N) | This study |  |  |
|  |  | INS-20-gRNA(N)-linker | TGAAAGGTTAAGTGTTGGGGAATAGTACTATTTAATCGCGTTTTAGAGCTAGAAATAG | | Building pACT1:Cas9-GFP, U6:sgINS-20(N) |  |  |  |
|  |  | pU6-gibson-F | ACACAGGAAACAGCTATGACCCAAATTTCTCCAACCAGTC | | Amplify U6:sgINS19 cassette (848 bp) for building pCRISPR-dual-INS19 by Gibson assembly | [10] |  |  |
|  |  | pSp6-ginbson-R | GCAAGCTTGGCGTAATCATGGACGGCCAGTGAATTCG | |  |  |  |  |
|  |  | Hind-F | CATGA TTACG CCAAG CTT | | Primer set used to amplify ACT1:Cas9-GFP, U6:sgINS19 cassette (9983 bp) for building pACT1:Cas9-GFP, dual, U6:sgINS19-KO by Gibson assembly | [10] |  |  |
|  |  | M13-R | GTCAT AGCTG TTTCC TGTG | |  |  |  |  |

Table S1. Oligonucleotides used in this study. (continued)

| **Usage** | | | **Oligo name** | **Sequence (5’ – 3’)** | **Specific purpose** | **Source** | |
| --- | --- | --- | --- | --- | --- | --- | --- |
| **IId-Mutation or knockout of domains** | INS-20-5’UTR-F2 | | AGTGAATTCGAGCTGAGCTCCCGCCTTAAAGTCAAATAGATTGTAC | Amplify the full length and part of the 5'UTR of INS-20 (3120 bp) | This study |  |  |
|  | INS-20-C-R | | AGCCCGAGCCCTTGCTAGCCTCATCTTTCATGAATTTGAGTTGGTTATATGC |  |  |  |  |
|  | INS-20-Nter-F | | CCAGGAAAGCATACTTATGGTAATGAAACTTTTGAACTAGAGCATAAAT | Eliminate the N-terminal structural domain in the donor plasmid of INS-20 | This study |  |  |
|  | INS-20-Nter-R | | CATAAGTATGCTTTCCTGGCCTGCTAATAAGGAAAACATCAAG |  |  |  |  |
|  | INS-20-Cter-F | | GCTAGCAAGGGCTCGGGCTCGAC | Eliminate the C-terminal structural domain in the donor plasmid of INS-20 | This study |  |  |
|  | INS-20-Cter-R | | AGCCCGAGCCCTTGCTAGCTGGAGCCAGGCTATCTATAGAAAAAAAATTAAGATC |  |  |  |  |
|  | INS-20-M-F | | GCAGCTGCGGCAGCCGCTTCAATATTTATTAACACAAAAAAATACCCGGAAATTTAC | Mutate the active site (HLLEQ) of INS-20 | This study |  |  |
|  | INS-20-M-R | | AGCGGCTGCCGCAGCTGCTATTCCAGGAATATATTCGG |  |  |  |  |
| **IId-GFP** | 3'tk gRNA(C)-linker | | CTATTTCTAGCTCTAAAACATAAGAATTTGTTCGCCAGACCCCAACACTTAACCTTTCA | Building pACT1:Cas9-GFP, U6:sgTK(C) | This study |  |  |
|  | TK-control-CF | | AGTGAATTCGAGCTGAGCTCTTACATTTCAAGTATCTGAAAATGAACCCTTTTGG | Amplify TK C-terminus homology arm (896 bp) | This study |  |  |
|  | control-TK-CR | | AGCCCGAGCCCTTGCTAGCGAAATTGTATTCTTCACAATTAATTATATGATG |  |  |  |  |
|  | linker-F | | GCTAGCAAGGGCTCGGGCTCGAC | Amplify RIBO-3'UTR (336 bp) | This study |  |  |
|  | ribo-R | | TTAAGCAAAGAAACTTATGAAAACTGTATTACC |  |  |  |  |
|  | pActin-F | | TCATAAGTTTCTTTGCTTAATCAGAATGAGTTGGTTATAAACAGTAATAATAG | Amplify GFP tag (2297 bp) | This study |  |  |
|  | Actin-3UTR-R | | ATATTTAGTTTCCCCACCTGGTATATCCGCCTGTCACG |  |  |  |  |
|  | peno-F | | AGGTGGGGAAACTAAATATACTGAAATTCG | Amplify Nluc-neo selection cassette (1751 bp) | This study |  |  |
|  | neo-R | | AATTAATCAGAAGAATTCGTCAAGAAG |  |  |  |  |
|  | TK-3UTR-F | | GACGAATTCTTCTGATTAATTAAGGC | Amplify plasmid backbone and TK 3'UTR homology arm (3637 bp) | This study |  |  |
|  | Laz R2 | | GAGCTCAGCTCGAATTCAC |  |  |  |  |

Table S1. Oligonucleotides used in this study. (continued)

| **Usage** | **Oligo name** | **Sequence (5’ – 3’)** | **Specific purpose** | **Source** | |
| --- | --- | --- | --- | --- | --- |
| **IId-PCR  genotyping** | 18S-LC2-F2 | AAGTATAAACCCCTTTACAAGTA | Quantify oocysts number by qPCR | [31] |  |
|  | 18S-LC2-R2 | TATTATTCCATGCTGGAGTATTC |  |  |  |
|  | INS-19-1-Tag-5-F | AGAAGGTGGTCATCCAATAGTAAATCTTATTCAATGTA | Primer set “5’ Ins”, WT = null; INS-19-3HA tagging = 1364 bp | This study |  |
|  | INS-19-1-Tag-5-R | CCACTAATTCATATTCCCGCCCTTAGAG |  |  |  |
|  | INS-19-1-Tag-3-F | GCTGAAGAACTTGGTGGTGA | Primer set “3’ Ins”, WT = null; INS-19-3HA tagging or KO = 854 bp | This study |  |
|  | INS-19-1-Tag-3-R | GAGTGAAATAATGATGATGTAAAAGATTGTAATGACTT |  |  |  |
|  | INS-19-2-Tag-5-F | AAATGTTGAGTAGAATCTTTGATCTAGACTTGTTGCCAGA | Primer set “5’ Ins”, WT = null; INS-20-3HA tagging = 1324 bp | This study |  |
|  | INS-19-2-Tag-5-R | CCACTAATTCATATTCCCGCCCTTAGAG |  |  |  |
|  | INS-19-2-Tag-3-F | GCTGAAGAACTTGGTGGTGA | Primer set “3’ Ins”, WT = null; INS-20-3HA tagging, KO, INS-20-Nter-KO, INS-20-Cter-KO = 1557 bp | This study |  |
|  | INS-19-2-Tag-3-R | TTTCCAGGTACGAGTGTTTGATATTTAG |  |  |  |
|  | INS-19-1-KO-5-F | ATATATTATTTCTTGATTATTGTCAAAATAAATTGAATA | Primer set “5’ Ins”, WT = null; ΔINS-19= 448 bp | This study |  |
|  | INS-19-1-KO-5-R | CCACTAATTCATATTCCCGCCCTTAGAG |  |  |  |
|  | cds-INS-19-1-F | AGAAGGTGGTCATCCAATAGTAAATC | WT = 625 bp; ΔINS-19 = null | This study |  |
|  | cds-INS-19-1-R | GCCAGCTGTTAAAATGCCCA |  |  |  |
|  | INS-19-2-KO-5-F | GACTGGAAATTATTGATTTTAATGAGTTC | Primer set “3’ Ins”, WT = null; ΔINS-20 = 1859 bp | This study |  |
|  | INS-19-2-KO-5-R | CCACTAATTCATATTCCCGCCCTTAGAG |  |  |  |
|  | cds-INS-19-2-F | AGGTGGTCACACAATAGTCG | WT = 638 bp; ΔINS-20 = null | This study |  |
|  | cds-INS-19-2-R | AATAATTTAATAGAAAACCAACTGTCATGATGTCCT |  |  |  |
|  | INS-20-Nter-KO-5-F | GACTGGAAATTATTGATTTTAATGAGTTC | Primer set “5’ Ins”, WT = null; ΔINS-20-Nter = 5243 bp | This study |  |
|  | INS-20-Nter-KO-5-R | CCACTAATTCATATTCCCGCCCTTAGAG |  |  |  |

Table S1. Oligonucleotides used in this study. (continued)

| **Usage** | **Oligo name** | **Sequence (5’ – 3’)** | **Specific purpose** | **Source** |
| --- | --- | --- | --- | --- |
| **IId-PCR  genotyping** | INS-19-2-Nter-cds-F | GACTGGAAATTATTGATTTTAATGAGTTC | WT = 2593 bp; ΔINS-20 = 2194 bp | This study |
|  | INS-19-2-Nter-cds-R | GCAGTTGTTTATTTTGAATCTTTGA |  |  |
|  | INS-20-Cter-KO-5-F | GACTGGAAATTATTGATTTTAATGAGTTC | Primer set “5’ Ins”, WT = null; ΔINS-20-Cter = 4337 bp | This study |
|  | INS-20-Cter-KO-5-R | CCACTAATTCATATTCCCGCCCTTAGAG |  |  |
|  | INS-19-2-Cter-cds-F | GATAATTATACAAAAATTAAATACTATATCGCTGACATTG | WT = 1003 bp; ΔINS-20 = null | This study |
|  | INS-19-2-Cter-cds-R | CGTACGTTGAATAAAATCGACTATTGTG |  |  |
|  | WT-Control-F | ACTTTGAGTGGGAGGAAG | Primer set “Control” used to detect INS3 insertion site (359 bp) | [10] |
|  | WT-Control-R | AGCGAGTCAGATACAAGC |  |  |
